# Supplementary material for: Evaluating a Novel Treatment Adapting a Cognitive Behaviour Therapy Approach for Sexuality Problems after Traumatic Brain Injury: A Single Case Design with Nonconcurrent Multiple Baselines
Source: J Clin Med. 2022 Jun 19;11(12):3525. doi: 10.3390/jcm11123525 (PMC9225377; doi:10.3390/jcm11123525)
Supplement: Supplementary file 1 [file jcm-11-03525-s001.zip › jcm-1721342-supplementary.pdf]

Supplementary Table S1. The Risk of Bias in N of 1 Trials scale.

RoBiNT Scale			
**Internal validity (IV) scale**			
**Item**	**Points**	**Criteria**	**Score**
1. Design with control	2 points:<br><br><br>1 point:<br>0 points:	At minimum ABAB with 4 phases; concurrent multiple baseline design with six phases, 3 tiers; alternating treatment design with sets of alternating sequences; changing criterion design with 4 steps; for medical N of 1 3x AB pairs<br>ABA or 3phase variant; MBD with 4-5 phases; ATD with <3 sets of alternating sequences<br>AB or AB with follow up, **non-concurrent MBD**	**0**
2. Randomisation	2 points<br>1 point:<br>0 points:	**Randomise: sequence and/or onset for all phases**<br>Restricted randomisation; counterbalancing<br>No information; randomisation of other aspects of the study	**2**
3. Sampling of behaviour	2 points:<br>1 point:<br>0 points:	**5 or more points in every phase**<br>At least 3 points in every phase<br><3 data points in any phase	**2**
4. Blinding of people involved in intervention	2 points:<br>1 point:<br>0 points	Double blinding of participant and practitioner<br>Participant or practitioner blinded<br>**Neither participant nor practitioner are blinded**	**0**
5. Blinding of Assessors	2 points:<br><br>1 point<br>0 points	Assessors blind to all phases; use of computer/machine free from human involvement, outcomes self-reported and participant is blind<br>**Independent assessor(s) but not blind to phase**<br>Neither participant nor practitioner are blind to phase	**1**
6. Interrater agreement	2 points<br>1 point<br>0 points	Machine degenerated data<br>A reasonably objective measure used or agreement is less than or equal to 70%<br>Agreement is <70%; **subjective measures used**; consensus ratings alone	**0**
7. Treatment Adherence	2 points:<br><br><br>1 point<br><br>0-point	Machine delivered intervention or adherence assessed (i)against clear rating system, (ii)assessor is independent of practitioner/patient (iii) >20% of data is sampled (iv) resulting in >80% adherence<br>**Adherence meets 2/4 criteria above and includes (a) assessor independent to practitioner and (b) adherence >70%**<br>Adherence <70%, assessor not independent of patient, components only loosely related to adherence	**1**

External validity (EV) scale			
**Item**	**Points**	**Criteria**	**Score**
8. Baseline characteristics	2 points:<br><br>1 point<br><br>0 points:	Analysis of baseline characteristics and age, sex, aetiology and severity of condition and the way they inform the intervention<br>**The provision of demographic, medical and functional status variables, or a clinical profile of test scores**<br>No analysis of baseline condition or incomplete listing of participant characteristics	**1**

9. Setting	2 points:<br><br>1 point:<br>0 points:	**Description of the general location and detailed description of the specific environment**<br>Description of either general location or specific environment but detail are sparse<br>Neither are provided	**2**
10. DV (target behaviour)	2 points:<br><br>1 point:<br><br>0 points:	**Target b/h is operationalised in precise terms and the methods of measurement are described**<br>Target b/h is operationally described but description and or method of measurement is not clear or precise<br>Target b/h is not operationally defined	**2**
11. IV	2 points:<br><br>1 point:<br>0 points:	**Detailed description of content of intervention including any equipment/manuals and 3 procedural details: number, duration and frequency of sessions**<br>General description of intervention, and 2/3 procedural details<br>Intervention described only in general terms, <2/3 details	**2**
12. Raw data record	2 points:<br><br>1 point:<br><br><br>0 points:	**Raw data record w data point for every session. If trials>10, raw data for 3 or more cases**<br>If trials>10 raw data for less than 3 cases, complete raw data for 2 participants, or provision of data record but data is aggregated or averaged or provision of data record but a prior decision not to record data for every session.<br>No raw data; omitted data, data only reported for select phases.	**2**
13. data analysis	2 points:<br><br>1 point:<br><br><br>0 points:	**Systematic VA with specific protocol or VA aided by quasi-statistical methods or statistical analysis with rational.**<br>Systematic/aided VA with selection of analytic techniques or statistical analysis w no rational or prior decision re the level of the target b/h consisting of an empirically derived clinically meaningful change<br>VA without data analysis; analysis note conducted on target b/h, arbitrary selection of level of target b/h.	**2**
14. Replication	2 points:<br>1 point:<br>0 points:	**1 original + 3 replications**<br>1 original + 1 or 2 replications<br>No replication	**2**
15. Generalisation	2 points:<br>1 point:<br>0 points:	Specific generalisation measure probed in every phase<br>**Specific generalisation measure probed in at least pre and post treatment phases**<br>No generalisation measure	**1**
INTERNAL TOTAL	6/14		
EXTERNAL TOTAL	14/16		
TOTAL	20/30		

Supplementary Table S2. Participants' fatigue, pain interference, and independence baseline raw scores.

Participant	FSS	TBI-QOL Pain Interference	TBI-QOL Independence
AA	41	12	39
BB	59	38	38
CC	22	24	36
DD	58	21	19
EE	62	43	16
FF	53	29	26
GG	34	29	28
HH	36	18	24
II	27	10	37

FSS, Fatigue Severity Scale; TBI-QOL, Traumatic Brain Injury – Quality of Life.
